# Spectral estimation in unevenly sampled space of periodically expressed microarray time series data

**DOI:** 10.1186/1471-2105-8-137

**Published:** 2007-04-24

**Authors:** Alan Wee-Chung Liew, Jun Xian, Shuanhu Wu, David Smith, Hong Yan

**Affiliations:** 1 School of Information & Communication Technology, Griffith University, Brisbane, Australia; 2 Department of Electronic Engineering, City University of Hong Kong, Hong Kong; 3 Department of Mathematics, Sun Yat-sen University, Guangzhou, 510275, China; 4 School of Computer Science and Technology, Yantai University, Yantai, 264005, China; 5 Department of Biochemistry, University of Hong Kong, Pok Fu Lam, Hong Kong; 6 School of Electronic and Information Engineering, University of Sydney, NSW2006, Australia

## Abstract

**Background:**

Periodogram analysis of time-series is widespread in biology. A new challenge for analyzing the microarray time series data is to identify genes that are periodically expressed. Such challenge occurs due to the fact that the observed time series usually exhibit non-idealities, such as noise, short length, and unevenly sampled time points. Most methods used in the literature operate on evenly sampled time series and are not suitable for unevenly sampled time series.

**Results:**

For evenly sampled data, methods based on the classical Fourier periodogram are often used to detect periodically expressed gene. Recently, the Lomb-Scargle algorithm has been applied to unevenly sampled gene expression data for spectral estimation. However, since the Lomb-Scargle method assumes that there is a single stationary sinusoid wave with infinite support, it introduces spurious periodic components in the periodogram for data with a finite length. In this paper, we propose a new spectral estimation algorithm for unevenly sampled gene expression data. The new method is based on signal reconstruction in a shift-invariant signal space, where a direct spectral estimation procedure is developed using the B-spline basis. Experiments on simulated noisy gene expression profiles show that our algorithm is superior to the Lomb-Scargle algorithm and the classical Fourier periodogram based method in detecting periodically expressed genes. We have applied our algorithm to the Plasmodium falciparum and Yeast gene expression data and the results show that the algorithm is able to detect biologically meaningful periodically expressed genes.

**Conclusion:**

We have proposed an effective method for identifying periodic genes in unevenly sampled space of microarray time series gene expression data. The method can also be used as an effective tool for gene expression time series interpolation or resampling.

## Background

Periodic phenomena are widely studied in biology and there are numerous biological applications where periodicities must be detected from experimental biological data. Because the measured data are often non-ideal, efficient algorithms are needed to extract as much information as possible. Spectral estimation has been a classical research topic in digital signal processing and has recently found important applications in DNA microarray time series data analysis. Many spectral estimation methods have been proposed in the past decades, including the modified periodogram, the autoregressive (AR) model, the MUSIC algorithm and the multitaper method [1,2]. Although all these algorithms have their own advantages, they were all developed based on a basic assumption: the input signal is evenly sampled. However, in many real-world applications, the data can be unevenly sampled. For example, in DNA microarray gene expression experiments, a time series may be obtained with different sampling intervals [3-5]. Furthermore, an evenly sampled time series may contain missing values due to corruption or absence of some expression measurements [6,7]. A time series with missing values can be considered as an unevenly sampled time series in general.

Recently, several methods for detecting periodic gene expression have been proposed [8-15]. Lu et al. [8] have proposed a periodic-normal mixture (PNM) model to fit transcription profiles of periodically expressed genes in cell cycle microarray experiments. Ahdesmäki et al. [9] proposed a general-purpose robust testing procedure for finding periodic sequences in multiple time series data, which is based on a robust spectral estimator that is incorporated into a hypothesis testing framework using the so-called G-statistic together with correction for multiple testing. Chen [10] proposed a statistical inference approach, the C&G procedure, to effectively detect statistically significant periodically expressed genes based on two statistical hypothesis testing procedures. Wichert et al. [11] proposed to use the average periodogram as an exploratory tool to detect the presence of possible periodic genes and give an exact statistical test to determine whether or not a sinusoid is presence. Luan and Li [12] proposed to use the shape-invariant model combined with a cubic B-spline estimation to model periodic gene expression profiles. Ruf [13] is one of the first to treat evenly sampled gene expression time series with missing values as unevenly sampled data for spectral analysis using the Lomb-Scargle periodogram. Bohn et al. [14] have used the Lomb-Scargle periodogram in their attempt to detect rhythmic components in the circadian cycle of the Crassulacean acid metabolism plants. Glynn et al. [15] also used the Lomb-Scargle periodogram to detect periodic patterns in unevenly spaced gene expression time series. The Lomb-Scargle periodogram produces better results on unevenly sampled data than the classical Fourier transform method since it weights the data on a "per point" basis instead of on a "per time interval" basis [16]. Lomb [17] proved that this periodogram is the same as the classical periodogram in the case of equally spaced data. However, since the Lomb-Scargle method assumes that there is a single stationary sinusoid wave with infinite support, it introduces spurious periodic components in the periodogram for data with a finite length. Also, due to the effect of noise in the data, it may produce inaccurate estimation results.

In this paper, we propose a new spectral estimation technique for unevenly sampled data. Our method models the signal in a shift-invariant signal space, for which many theories and algorithms are available [18-25]. In our method, a direct spectral estimation formula is derived based on the B-spline basis that has finite support. Experiments on simulated noisy periodic signals show that our algorithm is more accurate in detecting periodicity compared to the Lomb-Scargle algorithm.

## Results and discussion

Our method is based on signal reconstruction in a shift-invariant signal space, where a direct spectral estimation procedure is developed using the B-spline basis. The details of the reconstruction algorithm and the power spectrum density (PSD) estimation are given in the Method Section.

### Simulated data

We first test our spectral estimation algorithm on simulated signals to compare the estimation accuracy with the Lomb-Scargle method. A cosine curve has been used to represent the ideal expression of a gene that goes from an "on" state, to an "off" state, and then back to "on" [11]. For a gene *g *and expression level observed at time *t*_*i*_, we denote the time series by *Y*_*g*_(*t*_*i*_),

where Yg(ti)βcos⁡(tiπ12)+εg(ti)
 MathType@MTEF@5@5@+=feaafiart1ev1aaatCvAUfKttLearuWrP9MDH5MBPbIqV92AaeXatLxBI9gBaebbnrfifHhDYfgasaacH8akY=wiFfYdH8Gipec8Eeeu0xXdbba9frFj0=OqFfea0dXdd9vqai=hGuQ8kuc9pgc9s8qqaq=dirpe0xb9q8qiLsFr0=vr0=vr0dc8meaabaqaciaacaGaaeqabaqabeGadaaakeaacqWGzbqwdaWgaaWcbaGaem4zaCgabeaakiabcIcaOiabdsha0naaBaaaleaacqWGPbqAaeqaaOGaeiykaKccciGae8NSdiMagi4yamMaei4Ba8Maei4CamNaeiikaGYaaSaaaeaacqqG0baDdaWgaaWcbaGaeeyAaKgabeaakiab=b8aWbqaaiabigdaXiabikdaYaaacqGGPaqkcqGHRaWkcqWF1oqzdaWgaaWcbaGaee4zaCgabeaakiabcIcaOiabdsha0naaBaaaleaacqWGPbqAaeqaaOGaeiykaKcaaa@4B11@

for *i *= 1,...,*N *and *g *= 1,...,*G*. Our test data consists of simulated time series data for the expression of *G *= 1000 genes, where 900 of them are random genes (i.e., *β *= 0) and 100 are noisy periodic genes.

To obtain this dataset, the time series (genes) is first evenly sampled at 48 points. That is, *t*_*i *_= *i*, (*i *= 1,...,48). Then, time points are randomly deleted from each time series to simulate the uneven sampling situation.

**Figure 1:**
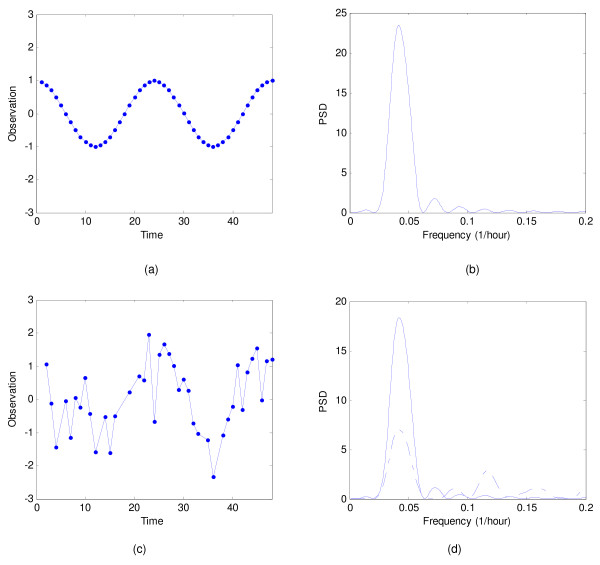
**Simulated data**. Comparison of spectral estimation for simulated data: (a) simulated cosine signal with even sampling (*N *= 48), (b) the periodogram of the simulated signal in (a) obtained using the Fourier transform, (c) simulated noisy cosine signal with uneven sampling, (d) the periodogram of the signal in (c) obtained using the Lomb-Scargle method (dashed line), and the periodogram of the signal in (c) obtained using our method (solid line).

Figure 1(a) shows a simulated expression of a gene that has a 24-hour period with data samples taken every half-hour. Its corresponding periodogram (see Figure 1(b)) shows a peak at the frequency of 1/24 Hz. Figure 1(c) shows the same cosine signal, but it is now corrupted with Gaussian noise and unevenly sampled. The periodograms, obtained using the Lomb-Scargle method and our algorithm, are shown in Figure 1(d). The peak frequencies in the periodograms obtained using the Lomb-Scargle method and our method are 1/22 Hz and 1/24 Hz, respectively. Clearly our method is more accurate than the Lomb-Scargle algorithm. Our method also produces fewer and smaller false peaks in the spectrum.

**Figure 2:**
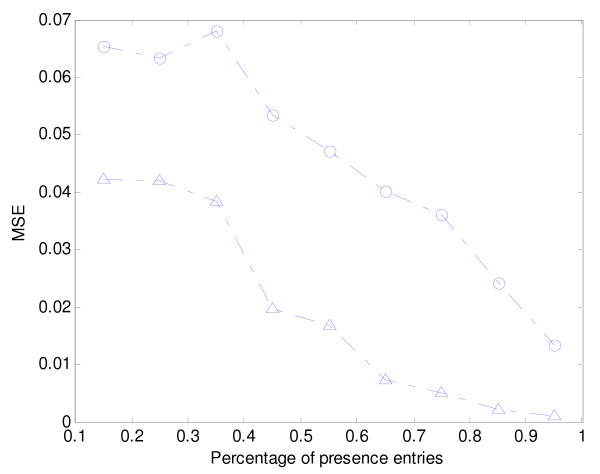
**Dominant frequency errors for simulated data**. Comparison of dominant frequency errors of spectral estimation for simulated data. MSE of dominant frequencies of spectral estimation obtained using the Lomb-Scargle method (-○-), and our method (-△-) according to various percentages of presence entries are shown.

In the second simulation test, we use the 100 simulated noisy periodic gene profiles and compare our method with the Lomb-Scargle approach in terms of errors in the dominant frequency. In Figure 2, we show the mean-square error in dominant frequency under various percentages of presence entries. From Figure 2, we observe that our method is better than the Lomb-Scargle method.

Finally, we use the entire 1000 simulated genes and the false discovery rate (FDR) gene selection strategy using G-statistic to test the accuracy and sensitivity of our method. Lomb-Scargle's test is used for Lomb-Scargle method. We added artificial Gaussian noise with mean *μ *= 0 and various SD values (*σ *= 0.01, 0.2 and 0.6). In Table 1, we compare the effectiveness of the two methods in detecting periodic gene expressions with different missing ratios and various noise levels under the same False Discovery Rate *q*. From Table 1, we find that our method is better than the Lomb-Scargle method in detecting periodic expressions with various missing ratios. Our method is also more robust than the Lomb-Scargle method under different noise levels.

**Table 1:** Number of periodic genes detected using different methods for simulated data

		P	
		
Noise levels (*σ*)	Missing ratio	Lomb-Scargle method	Our Algorithm
0.01	15%	93	98
	20%	88	92
	40%	68	80
	65%	40	65
0.2	15%	85	92
	20%	75	85
	40%	60	78
	65%	32	50
0.6	15%	72	89
	20%	65	80
	40%	51	65
	65%	28	47

The simulated data consists of 1000 time series (genes). The total number of periodic genes is 100. P is the number of periodic genes that are statistically significant for a FDR level of q = 0.02. The number of false positives FP can be computed as FP = q*TP/(1-q), where TP is the number of true positives. Keeping q at a fixed level ensures that we do not sacrifice specificity for sensitivity.

### Experimental data

#### Plasmodium falciparum

**Figure 3:**
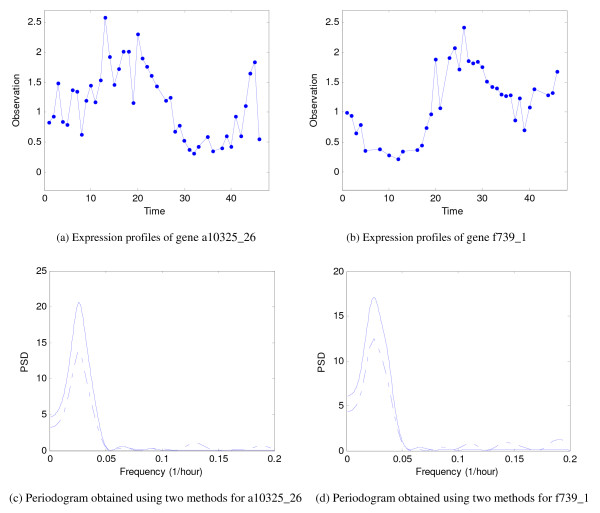
**Plasmodium falciparum data**. The expression time series of (a) gene a10325_26 and (b) gene f739_1 in the Plasmodium falciparum microarray dataset. The periodogram obtained using (a) the Lomb-Scargle method (dashed line) and our algorithm (solid line).

We have tested our algorithm on the gene expression data of Plasmodium falciparum, which is one of the species that cause human malaria [27]. The gene expression time series from the asexual intraerythrocytic developmental cycle (IDC) of Plasmodium falciparum are strongly periodic. Identifying periodically expressed genes is useful for understanding the genome of Plasmodium falciparum and designing effective vaccines for prevention of human malaria. In the gene expression database from Bozdech et al. [27], data values at the 23rd and the 29th hours are completely missing. An example of a gene expression profiles from the database is shown in Figure 3(a), and its periodograms obtained by using the Lomb-Scargle algorithm and our algorithm are shown in Figure 3(c). The frequencies corresponding to the peaks in the periodograms obtained by using the Lomb-Scargle method and our method are 1/43.10 Hz and 1/44.13 Hz, respectively. Another example is shown in Figure 3(b). The frequencies corresponding to the peaks in the periodograms obtained by using the Lomb-Scargle method and our method are 1/48.68 Hz and 1/47.85 Hz, respectively (see Figure 3(d)). We can see from these diagrams that our algorithm can effectively reduce the spurious oscillation components in the spectra.

**Figure 4:**
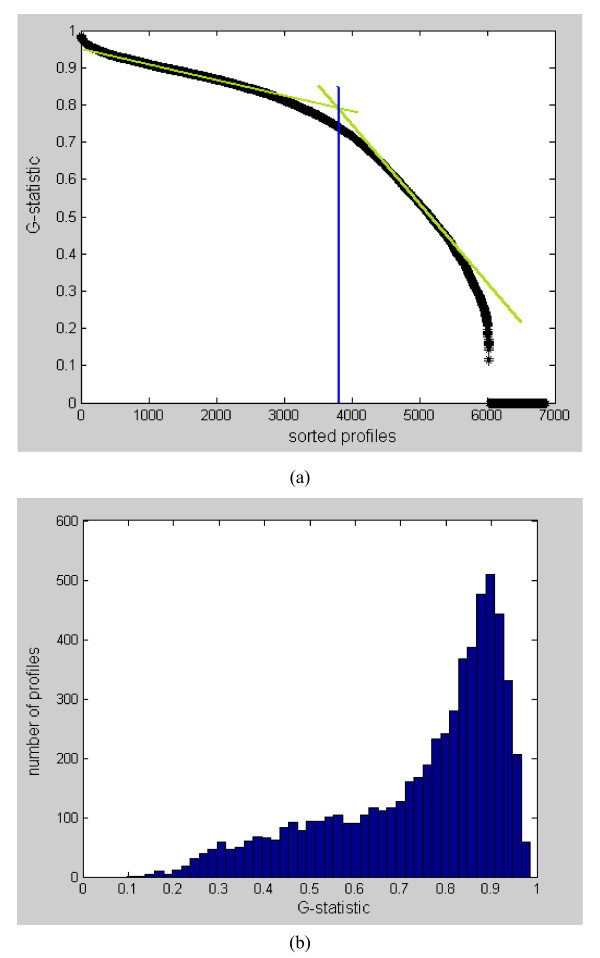
**(a)-(b). G-statistic values of P. falciparum**. (a) Sorted G-statistic values. There is a change in the trend of the ranked G-statistic values at around the 4000 sorted profiles, indicating that two classes of profiles, i.e., periodic/aperiodic, are present in the dataset. Nevertheless, this classification is not exact as there is not distinctive sharp drop in the ranked G-statistics. (b) Histogram of the G-statistic values of P. falciparum. A profile with larger G-statistic value implies it is much more likely to be periodic. We see that many of the profiles are periodic. However, no distinct valley can be observed, indicating that it is difficult to set a cut-off between periodicity and aperiodicity here.

**Figure 5:**
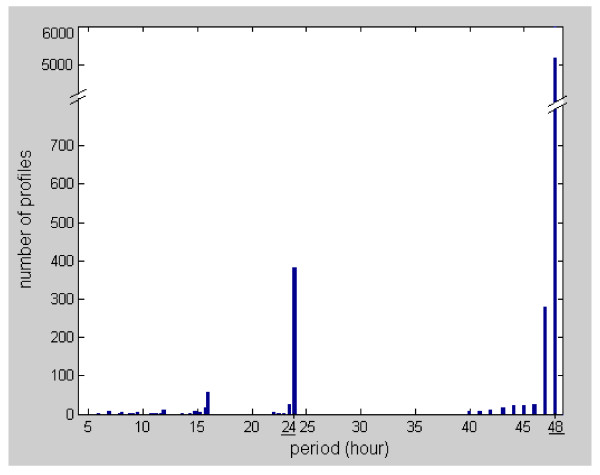
**Histogram plot of the dominant period of P. falciparum**. The histogram plot shows a prominent period of 48 hours with a count of around 5000 profiles.

**Figure 6:**
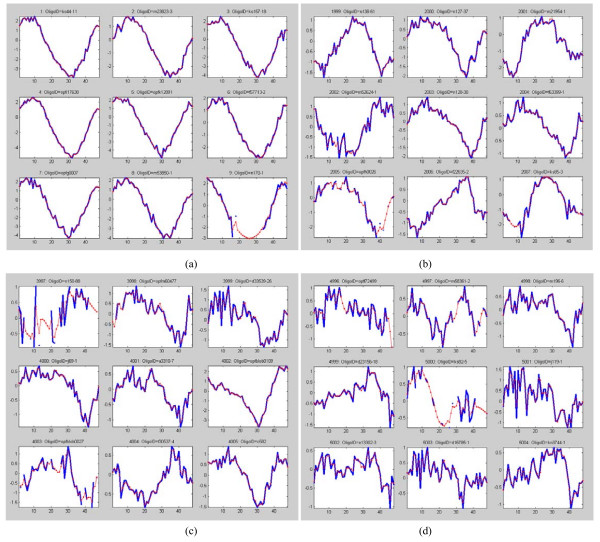
**(a)-(d). Expression profiles of P. falciparum at different ranking**. (a) Top 9 ranking profiles, (b) profiles that ranked at around 2000, (c) profiles that ranked at around 4000, (d) profiles that ranked at around 5000.

The Plasmodium falciparum dataset was analyzed by Bozdech et al. [27] using the fast Fourier transform (FFT), and later by Glynn et al. [15] using the Lomb-Scargle algorithm. Bozdech et al. [27] identified 3719 periodic genes in the Quality Control dataset of Plasmodium falciparum, while Glynn et al. [15] found 4355 periodic genes in the complete dataset of 6875 (excluding those profile with unknown oligo ID). Our analysis has shown that the number of periodic profiles in the complete dataset should be around 3700 to 4000. Our estimate is based on analyzing the trend of the sorted G-statistic (computed using Equation (14) in Method) as shown in Figure 4(a). The intersection of the two distinct slopes points indicates a sudden change in the G-statistic trend. Nevertheless, no distinct cut-off between periodic/aperiodic profiles can be identified here. Figure 4(b) shows the histogram plot of the G-statistic. Note that a profile with larger G-statistic value implies it is much more likely to be periodic. We see that many of the profiles are likely to be periodic. No distinct valley can be observed in the histogram, indicating that a cut-off for periodicity/aperiodicity is difficult to be obtained here. We also examine the periods of the profiles, and Figure 5 shows the histogram plot. It can be seen that there is a prominent period of 48 hours with a count of around 5000 profiles. This result agreed largely with that of Bozdech et al. [27] (see their Figure 2 that discusses the P. Falciparum IDC Transcriptome phases with a period of roughly 48 hours) and that of Glynn et al. [15] (see the histogram of period values in their Supplementary). Nevertheless, our result also shows a much less prominent peak at a period of 24 hours with a low count of around 400 profiles. In Figure 6(a), we show the top 9 ranking profiles, they can be seen to be highly periodic. In Figure 6(b)–(d) , we show the profiles that ranked around 2000, 4000, and 5000, respectively. Even at around a ranking of 5000, some profiles (e.g. see profile at rank 4997) can still be judged to be somewhat periodic. We provide a ranked list of the 6875 profiles in Additional File 1.

#### Yeast

Spectral analysis is useful for the identification of cell-cycle-regulated genes. Spellman et al. [3] monitored genome-wide mRNA levels for 6178 yeast ORFs simultaneously using several different methods of synchronization including an *α *(alpha)-factor-mediated G1 arrest which covers approximately two cell-cycle periods with measurements at 7 min intervals for 119 min with a total of 18 time points, a temperature-sensitive cdc15 mutation to induce a reversible M-phase arrest, and a temperature-sensitive cdc28 mutation to arrest cells in G1 phase reversibly, and finally, an elutriation synchronization to produce the elutriation dataset of 14 time points (raw data available at [35]). For the cdc15 experiment, gene expression data were measured every 10 min for 290 min, lacking observations for the 20, 40, 60, 260 and 280 min time points, and gives a total of 24 time points. For the cdc28 experiment, samples were taken every 10 min from 0 to 160 min for a total of 17 time points. These four microarray datasets have spawned a large body of work on the gene expressions of the yeast cell cycle.

**Figure 7:**
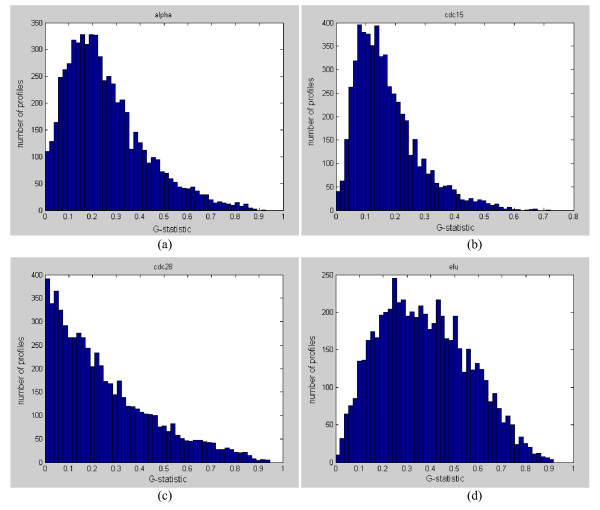
**(a)-(d). Histogram distributions of G-statistic values for the four yeast datasets**. (a) G-statistic histogram for the alpha dataset, (b) G-statistic histogram for the cdc15 dataset, (c) G-statistic histogram for the cdc28 dataset, and (d) G-statistic histogram for the elutriation dataset. In each dataset, there is a continuum of distribution and a clear cutoff for periodicity/aperiodicity cannot be identified.

**Figure 8:**
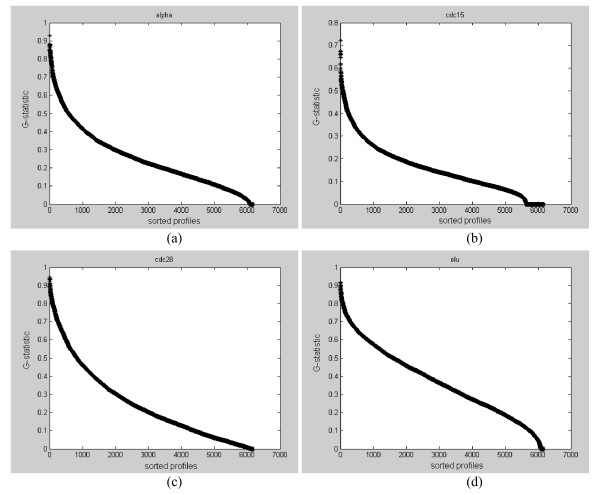
**(a)-(d). The sorted G-statistic plots for the four yeast datasets**. (a) Sorted G-statistic for the alpha dataset, (b) sorted G-statistic for the cdc15 dataset, (c) sorted G-statistic for the cdc28 dataset, and (d) sorted G-statistic for the elutriation dataset.

Spellman [3] originally identified a total of 800 cell-cycle genes in all four datasets, while Wichert et al. [11] claimed 468 cyclic genes in alpha, 766 cyclic genes in cdc15, 105 in cdc28, and 193 in elutriation by using G-statistic as the test statistic. Chen [10] detected 473 cyclic genes in alpha, 788 cyclic genes in cdc15, 27 in cdc28, and 769 in elutriation by using the same G-statistic as the test statistic under the same FDR threshold level. However, we found that the fisher p-value (from Equation (13)) computed using the G-statistic has weak statistical power with such a short signal length. Instead, we analyze the four yeast datasets for periodicity and rank the gene expression profiles according to their G-statistic. Figure 7(a)–(d) show the histogram distributions of G-statistic values for the four datasets. We see that there is a continuum of distribution and a clear cutoff for periodicity/aperiodicity cannot be identified (no obvious valleys exist). The sorted G-statistic plots in Figure 8(a)–(d) also supported such an observation. Hence, a ranking of the gene expression profiles would be much more informative than just giving an ad-hoc estimate of the number of periodic genes (see Additional File 2 for a ranked list of all genes in each experiment).

**Figure 9:**
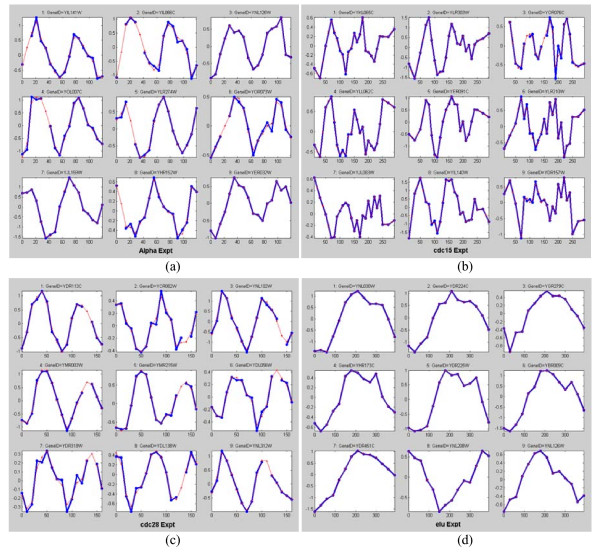
**(a)-(d). The top nine ranking genes in each of the four yeast datasets**. (a) Top 9 ranking genes for the alpha dataset, (b) top 9 ranking genes for the cdc15 dataset, (c) top 9 ranking genes for the cdc28 dataset, and (d) top 9 ranking genes for the elutriation dataset.

Figure 9(a)–(d) show the 9 top ranking genes in each of the four datasets. In Figure 9(a), we see that six of the nine highest-ranking expression profiles actually have missing values. Nevertheless, our algorithm was able to restore the missing values and rank the profiles properly. The top ranking profiles in the alpha and the cdc28 datasets can be observed to have two periods within the measurement intervals. This agrees well with the known estimated period of 55–77 minutes and 80–100 minutes for the alpha and cdc28 experiments, respectively. The top ranking expression profiles in the cdc15 dataset are much noisier compared to the top ranking profiles in the other three datasets. The profiles indicate a period of around 80–100 minutes, whereas the known estimated period for this dataset is around 60–80 minutes. The elutriation dataset result of (d) strongly indicated that the measurement interval is around one period (in agreement with many studies done on this dataset). For our top 9 ranking elutriation profiles in (d), 4 of them are within the top 9 ranking profiles in Wichert et al. [11] (see their Figure 6) and 1 is within the top 9 ranking profiles of Chen [10] (see their Figure 2).

As pointed out by Lichtenberg et al. [28], there is a remarkably poor agreement between the numbers of periodically expressed genes detected by various computational methods. To enable a more objective comparison between the performances of different algorithms, they proposed three benchmark sets B1, B2, and B3 (see their website [36]). Set B1 contains a total of 113 genes previously identified as periodically expressed in small-scale experiments. Set B2 contains 352 genes whose promoters were bound by at least one of the 9 known cell cycle transcription factors (i.e., Fkh1, Fkh2, Ndd1, Mcm1, Ace2, Mbp1, Swi4, Swi5, Swi6) in two Chromatin IP studies, and therefore many of the genes in this benchmark set should be expected to be cell cycle regulated. Set B3 contains 518 genes annotated in MIPS [37] as "cell cycle and DNA processing". However, since a large number of genes involved in the cell cycle are not subjected to transcriptional regulation (not periodic) and genes found in B1 were explicitly removed, only a small fraction of the genes in B3 are expected to be periodically expressed. They define a good method as one that is able to reproduce precious findings (B1), extract genes whose promoters are associated with known cell cycle transcription factors (B2), or enriched for genes that play a role in the cell cycle (B3).

**Figure 10:**
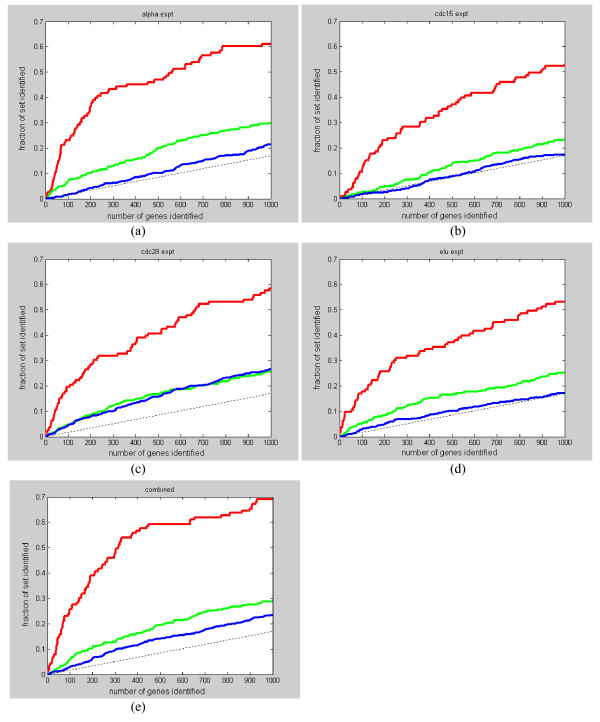
**(a)-(e). The fraction of benchmark sets B1, B2, and B3 recovered (i.e., coverage) as a function of gene rank for the four yeast datasets and the combined set**. (a) Coverage for the alpha dataset, (b) coverage for the cdc15 dataset, (c) coverage for the cdc28 dataset, (d) coverage for the elutriation dataset, and (e) coverage for the combined dataset. Red line is for benchmark set B1, green line is for benchmark set B2, blue line is for benchmark set B3, and dotted line is for random performance.

Figure 10(a)–(e) show the fraction of benchmark sets recovered (i.e., coverage) as a function of gene rank for the four datasets and the combined set (the G-statistic in the combined set is obtained by taking the maximum of the corresponding G-statistic values of the 4 datasets). The performance of all datasets is significantly better than random for B1 and B2. For B3, cdc15 and elutriation performances are close to random, whereas cdc28 performance is better than random. The superior performance of the combined dataset indicated that all experiments are valuable in yeast cell cycle study and should be considered together. Not surprisingly, the coverage for B3 is always less than B1 and B2 since only a small fraction of the genes in B3 are expected to be periodically expressed.

**Figure 11:**
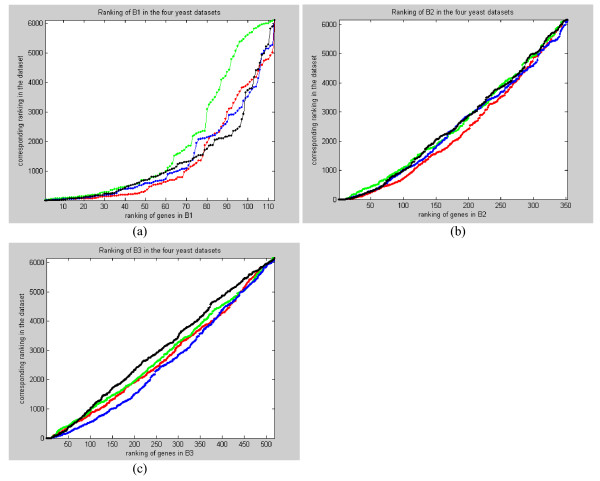
**(a)-(c). The ranking of the genes in the benchmark sets against the overall ranking in the four yeast datasets**. (a) Ranking in B1, (b) ranking in B2, and (c) ranking in B3. Red line is for the alpha dataset, green line is for the cdc15 dataset, blue line is for the cdc28 dataset, and black line is for the elutriation dataset. We see that many of the genes in the benchmark sets have no observable periodicity.

A detail investigation of the expression profiles in the benchmark sets shows that the generally low coverage is really due to the absence of periodicity in many of the profiles. In Figure 11(a)–(c) , we plotted the ranking of the genes in the benchmark sets against the overall ranking in the four datasets. We see that a large portion of the profiles in the benchmark sets is ranked very low in the four datasets. A close examination of these profiles confirms that they are nearly random with no observable periodicity. There could be two possible causes for this: (1) a large number of genes involved in the cell cycle are not directly transcription-regulated and therefore not periodic; (2) the genes are really periodic but experimental artifacts and noise has corrupted their profiles. Hence, for these profiles, it is expected that no algorithms would be able to identify them as periodic based on just a periodicity score.

**Figure 12:**
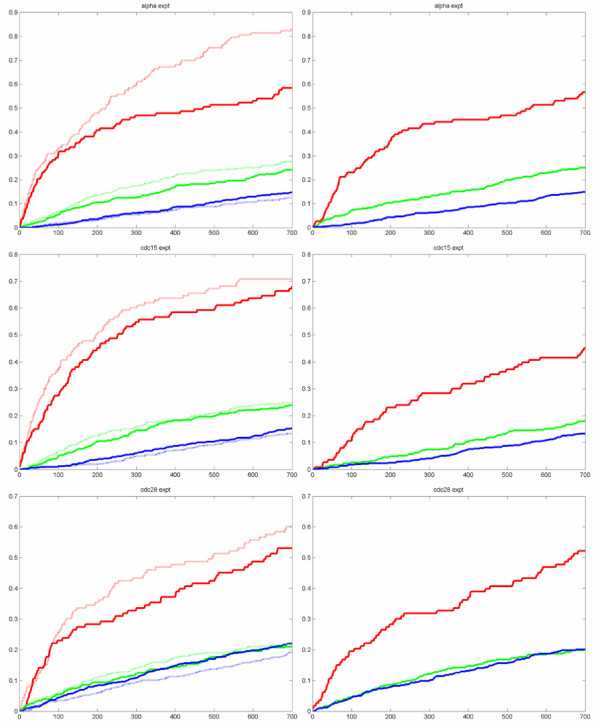
**Comparison of coverage as a function of gene rank between results of [28] and our method**. Left column: results of [28] on the benchmark sets B1, B2, and B3 when only periodicity is considered (solid lines), and when periodicity and regulation strength are both considered (dotted lines). Right column: results of our method on the benchmark sets B1, B2, and B3. Red line is for B1, green line is for B2, blue line is for B3. X-axis is the fraction of set identified, y-axis is the number of genes identified.

In [28], the strength of regulation (i.e., highly regulated genes have large standard deviation in their expression profiles) is an important criterion for the detection of periodically expressed genes. Regulation strength was not used as a criterion by our method (as well as the method of [11]) in detecting profile periodicity since the G-statistic value given by Equation (14) gives a normalized periodic score. Based on the periodicity criterion alone, our method has comparable performance with [28] for the alpha and cdc28 experiments. For the cdc15 experiment, our result is notably inferior (see Figure 12). However, if we look at the cdc15 profiles as shown in Figure 9, we see that even the high ranking profiles are very noisy, and hence the comparison for cdc15 have lower reliability with regard to periodicity behavior. When regulation strength is taken into consideration as well, the results of [28] clearly out-performance our results for the B1 dataset (see dotted curves in left column of Figure 12). This is not surprising since it was pointed out in [28] that the benchmark datasets B1 and B2 are biased towards periodic genes which are strongly regulated. In fact, it was observed in [28] that regulation strength alone outperforms pure periodicity score for the alpha factor experiment on both B1 and B2 datasets. However, for the B3 dataset, taking regulation strength into account actually gives inferior results for all three experiments. As noted by [28], the B3 dataset is likely to be biased toward small amplitude genes. This suggests that regulation strength is only helpful in situations where genes involved in the cell cycle are also significantly regulated. It would not be useful (and can in fact worsen the performance) in situations where genes are highly regulated but are not involved directly in cell cycle process.

**Figure 13:**
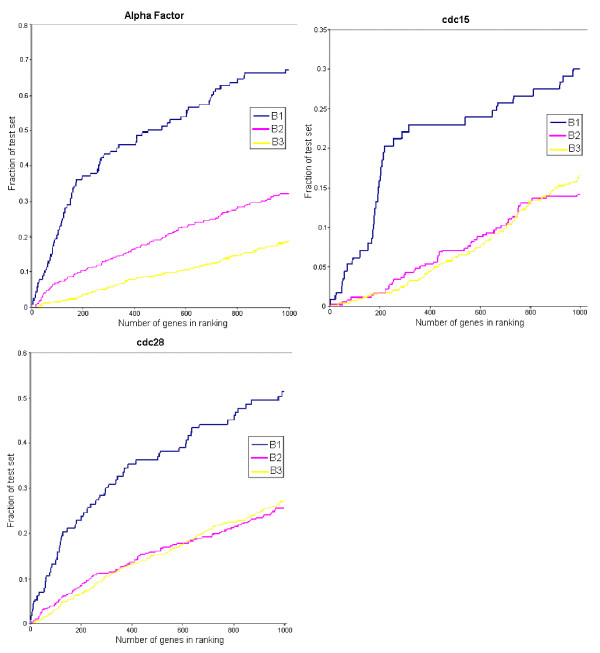
**Results of [29] on the benchmark sets B1, B2, and B3 (Figure adopted from [29])**. Our method (see Figure 10) compares favorably with that of [29]. Both the method of [29] and our method considered only periodicity as the sole criterion. We see that for the alpha factor experiment, our results are better for B3, comparable for B2, and inferior for B1. For the cdc15 experiment, our results are better for B1, B2, and B3. For the cdc28 experiment, our results are better for B1, while comparable for B2 and B3.

The three benchmark datasets are also analyzed in [29]. Both the method of [29] and our method considered only periodicity as the sole criterion. We cited their results in Figure 13. Comparing their results with our results as shown in Figure 10, we see that for the alpha factor experiment, our results is better for B3, comparable with theirs for B2, and inferior for B1. For the cdc15 experiment, our results are better for all three benchmark datasets. For this experiment, it is interesting to note that although our results are notably inferior to that of [28], they are significantly better than that of [29]. For the cdc28 experiment, our results are better for B1, while comparable for B2 and B3. The above comparative study indicates the difficulty in making general performance comparison between different algorithms even with benchmark datasets due to the differences in dataset characteristic.

## Conclusion

In this paper, we have proposed a new spectral estimation algorithm based on a signal reconstruction technique in an unevenly sampled space. The advantage of our algorithm over the Lomb-Scargle spectral estimation method is that the new algorithm can effectively reduce the effects of noise and spurious oscillation components and therefore improve the estimation accuracy. Experiments on simulated signals and real gene expression data show that our method is effective in identifying periodically expressed genes. Finally, we remark that this paper focuses on the improvement of periodicity estimation accuracy using spectral analysis algorithms. Another important issue is the statistical significance of the periodicity of a time series. Interested readers are referred to Chen [10], Wichert et al. [11] and Glynn et al. [15], who have used hypothesis testing to address this problem.

## Methods

### Mathematical model

In the following, we first review existing work on signal analysis in the shift-invariant signal space, and then derive the new spectral estimation algorithm.

Shannon's signal sampling and reconstruction theorem states that:

**Theorem**: If *f *∈ *B*_Ω _= {*f *∈ *L*^2 ^: supp f^
 MathType@MTEF@5@5@+=feaafiart1ev1aaatCvAUfKttLearuWrP9MDH5MBPbIqV92AaeXatLxBI9gBaebbnrfifHhDYfgasaacH8akY=wiFfYdH8Gipec8Eeeu0xXdbba9frFj0=OqFfea0dXdd9vqai=hGuQ8kuc9pgc9s8qqaq=dirpe0xb9q8qiLsFr0=vr0=vr0dc8meaabaqaciaacaGaaeqabaqabeGadaaakeaacuWGMbGzgaqcaaaa@2E11@ ⊂ [-*A*, *A*]} and 0 < 2*TA *≤ 1, then

f(x)=2TA∑n∈Zf(nT)sin⁡[2πA(t−nT)]2πA(t−nT)
 MathType@MTEF@5@5@+=feaafiart1ev1aaatCvAUfKttLearuWrP9MDH5MBPbIqV92AaeXatLxBI9gBaebbnrfifHhDYfgasaacH8akY=wiFfYdH8Gipec8Eeeu0xXdbba9frFj0=OqFfea0dXdd9vqai=hGuQ8kuc9pgc9s8qqaq=dirpe0xb9q8qiLsFr0=vr0=vr0dc8meaabaqaciaacaGaaeqabaqabeGadaaakeaacqWGMbGzcqGGOaakcqWG4baEcqGGPaqkcqGH9aqpcqaIYaGmcqWGubavcqWGbbqqdaGfqbqabSqaaiabd6gaUjabgIGiopXvP5wqSXMqHnxAJn0BKvguHDwzZbqegeKCPfgBaGabaiaa=PfaaeqaneaacqGHris5aaGccqWGMbGzcqGGOaakcqWGUbGBcqWGubavcqGGPaqkdaWcaaqaaiGbcohaZjabcMgaPjabc6gaUjabcUfaBjabikdaYGGaciab+b8aWjabdgeabjabcIcaOiabdsha0jabgkHiTiabd6gaUjabdsfaujabcMcaPiabc2faDbqaaiabikdaYiab+b8aWjabdgeabjabcIcaOiabdsha0jabgkHiTiabd6gaUjabdsfaujabcMcaPaaaaaa@6627@

where supp f^
 MathType@MTEF@5@5@+=feaafiart1ev1aaatCvAUfKttLearuWrP9MDH5MBPbIqV92AaeXatLxBI9gBaebbnrfifHhDYfgasaacH8akY=wiFfYdH8Gipec8Eeeu0xXdbba9frFj0=OqFfea0dXdd9vqai=hGuQ8kuc9pgc9s8qqaq=dirpe0xb9q8qiLsFr0=vr0=vr0dc8meaabaqaciaacaGaaeqabaqabeGadaaakeaacuWGMbGzgaqcaaaa@2E11@ = {*x *: f^
 MathType@MTEF@5@5@+=feaafiart1ev1aaatCvAUfKttLearuWrP9MDH5MBPbIqV92AaeXatLxBI9gBaebbnrfifHhDYfgasaacH8akY=wiFfYdH8Gipec8Eeeu0xXdbba9frFj0=OqFfea0dXdd9vqai=hGuQ8kuc9pgc9s8qqaq=dirpe0xb9q8qiLsFr0=vr0=vr0dc8meaabaqaciaacaGaaeqabaqabeGadaaakeaacuWGMbGzgaqcaaaa@2E11@(*x*) ≠ 0} and f^
 MathType@MTEF@5@5@+=feaafiart1ev1aaatCvAUfKttLearuWrP9MDH5MBPbIqV92AaeXatLxBI9gBaebbnrfifHhDYfgasaacH8akY=wiFfYdH8Gipec8Eeeu0xXdbba9frFj0=OqFfea0dXdd9vqai=hGuQ8kuc9pgc9s8qqaq=dirpe0xb9q8qiLsFr0=vr0=vr0dc8meaabaqaciaacaGaaeqabaqabeGadaaakeaacuWGMbGzgaqcaaaa@2E11@ is the Fourier transform of *f *defined by

f^(ω)=−∞+∞f(t)e−i2πωtdt.
 MathType@MTEF@5@5@+=feaafiart1ev1aaatCvAUfKttLearuWrP9MDH5MBPbIqV92AaeXatLxBI9gBaebbnrfifHhDYfgasaacH8akY=wiFfYdH8Gipec8Eeeu0xXdbba9frFj0=OqFfea0dXdd9vqai=hGuQ8kuc9pgc9s8qqaq=dirpe0xb9q8qiLsFr0=vr0=vr0dc8meaabaqaciaacaGaaeqabaqabeGadaaakeaacuWGMbGzgaqcaiabcIcaOGGaciab=L8a3jabcMcaPiabg2da9maavadabeWcbaGaeyOeI0IaeyOhIukabaGaey4kaSIaeyOhIukaoiabgUIiYdGccqWGMbGzcqGGOaakcqWG0baDcqGGPaqkcqWGLbqzdaahaaWcbeqaaiabgkHiTiabdMgaPjabikdaYiab=b8aWjab=L8a3jabdsha0baakiabdsgaKjabdsha0jabc6caUaaa@4B5B@

Equation (1) shows that the space of a bandlimited signal is identical to the space:

V(sinc)={f(x)=∑k∈Zcksinc(x−k):(ck)∈ℓ2}
 MathType@MTEF@5@5@+=feaafiart1ev1aaatCvAUfKttLearuWrP9MDH5MBPbIqV92AaeXatLxBI9gBaebbnrfifHhDYfgasaacH8akY=wiFfYdH8Gipec8Eeeu0xXdbba9frFj0=OqFfea0dXdd9vqai=hGuQ8kuc9pgc9s8qqaq=dirpe0xb9q8qiLsFr0=vr0=vr0dc8meaabaqaciaacaGaaeqabaqabeGadaaakeaacqWGwbGvcqGGOaakcqqGZbWCcqqGPbqAcqqGUbGBcqqGJbWycqGGPaqkcqGH9aqpcqGG7bWEcqWGMbGzcqGGOaakcqWG4baEcqGGPaqkcqGH9aqpdaGfqbqabSqaaiabdUgaRjabgIGiopXvP5wqSXMqHnxAJn0BKvguHDwzZbqegeKCPfgBaGabaiaa=PfaaeqaneaacqGHris5aaGccqWGJbWydaWgaaWcbaGaem4AaSgabeaakiabbohaZjabbMgaPjabb6gaUjabbogaJjabcIcaOiabdIha4jabgkHiTiabdUgaRjabcMcaPiabcQda6iabcIcaOiabdogaJnaaBaaaleaacqWGRbWAaeqaaOGaeiykaKIaeyicI4SaeS4eHW2aaWbaaSqabeaacqaIYaGmaaGccqGG9bqFaaa@657C@

Dowski et al. [30] have introduced a reconstruction formula for unevenly sampled signals that is a special case of Equation (2):

V={f:f(x)=∑n=0N−1cnsinc(x−n)}.
 MathType@MTEF@5@5@+=feaafiart1ev1aaatCvAUfKttLearuWrP9MDH5MBPbIqV92AaeXatLxBI9gBaebbnrfifHhDYfgasaacH8akY=wiFfYdH8Gipec8Eeeu0xXdbba9frFj0=OqFfea0dXdd9vqai=hGuQ8kuc9pgc9s8qqaq=dirpe0xb9q8qiLsFr0=vr0=vr0dc8meaabaqaciaacaGaaeqabaqabeGadaaakeaacqWGwbGvcqGH9aqpcqGG7bWEcqWGMbGzcqGG6aGocqWGMbGzcqGGOaakcqWG4baEcqGGPaqkcqGH9aqpdaGfWbqabSqaaiabd6gaUjabg2da9iabicdaWaqaaiabd6eaojabgkHiTiabigdaXaqdbaGaeyyeIuoaaOGaem4yam2aaSbaaSqaaiabd6gaUbqabaGccqqGZbWCcqqGPbqAcqqGUbGBcqqGJbWycqGGOaakcqWG4baEcqGHsislcqWGUbGBcqGGPaqkcqGG9bqFcqGGUaGlaaa@512E@

Since the sinc function has an infinite support and slow decay, it is seldom adopted in real applications. Xian et al. [24] found a good decay function that can replace the sinc basis function, but the new function still has an infinite support. To replace the sinc function with a general function *ϕ*, we first introduce a signal space that is called the shift-invariant (also called time-invariant) signal space:

V(φ)={f:f(x)=∑k∈Zckφ(x−k):(ck)∈ℓ2}
 MathType@MTEF@5@5@+=feaafiart1ev1aaatCvAUfKttLearuWrP9MDH5MBPbIqV92AaeXatLxBI9gBaebbnrfifHhDYfgasaacH8akY=wiFfYdH8Gipec8Eeeu0xXdbba9frFj0=OqFfea0dXdd9vqai=hGuQ8kuc9pgc9s8qqaq=dirpe0xb9q8qiLsFr0=vr0=vr0dc8meaabaqaciaacaGaaeqabaqabeGadaaakeaacqWGwbGvcqGGOaakiiGacqWFgpGzcqGGPaqkcqGH9aqpcqGG7bWEcqWGMbGzcqGG6aGocqWGMbGzcqGGOaakcqWG4baEcqGGPaqkcqGH9aqpdaGfqbqabSqaaiabdUgaRjabgIGiopXvP5wqSXMqHnxAJn0BKvguHDwzZbqegqvyO9wBHbaceaGaa4Nwaaqab0qaaiabggHiLdaakiabdogaJnaaBaaaleaacqWGRbWAaeqaaOGae8NXdyMaeiikaGIaemiEaGNaeyOeI0Iaem4AaSMaeiykaKIaeiOoaOJaeiikaGIaem4yam2aaSbaaSqaaiabdUgaRbqabaGccqGGPaqkcqGHiiIZcqWItecBdaahaaWcbeqaaiabikdaYaaakiabc2ha9baa@60C5@

where the coefficients {*c*_*i*_} are related to the choice of basis function, *ϕ*.

Signal reconstruction in the shift-invariant space is an active research area and there are many mathematical theories and algorithms available [18-25]. When the signal *f *∈ *V*(*ϕ*), we need to reconstruct signal *f *from sampled value {*f*(*x*_*i*_)}, where {*x*_*i*_} is the sampled point set. If {*x*_*i*_} is an evenly sampled point set, this problem can be regarded as signal reconstruction in an even sampled space. Otherwise, this is a signal reconstruction problem in an uneven sampled space.

In fact, the well-known autoregressive (AR) model can be regarded as a special case of signal reconstruction in the above signal space. For a given discrete data sequence *x*[*n*] for 0 ≤ *n *≤ *N *- 1, the sample at time index *n *is approximated by a linear combination of the previous *K *samples in the sequence based on the AR prediction model that can be written as,

x[n]=x˜[n]+e[n]=−∑k=1Kakx[n−k]+e[n],(n≥K),
 MathType@MTEF@5@5@+=feaafiart1ev1aaatCvAUfKttLearuWrP9MDH5MBPbIqV92AaeXatLxBI9gBaebbnrfifHhDYfgasaacH8akY=wiFfYdH8Gipec8Eeeu0xXdbba9frFj0=OqFfea0dXdd9vqai=hGuQ8kuc9pgc9s8qqaq=dirpe0xb9q8qiLsFr0=vr0=vr0dc8meaabaqaciaacaGaaeqabaqabeGadaaakeaafaqabeqacaaabaGaemiEaGNaei4waSLaemOBa4Maeiyxa0Laeyypa0JafmiEaGNbaGaacqGGBbWwcqWGUbGBcqGGDbqxcqGHRaWkcqWGLbqzcqGGBbWwcqWGUbGBcqGGDbqxcqGH9aqpcqGHsisldaGfWbqabSqaaiabdUgaRjabg2da9iabigdaXaqaaiabdUealbqdbaGaeyyeIuoaaOGaemyyae2aaSbaaSqaaiabdUgaRbqabaGccqWG4baEcqGGBbWwcqWGUbGBcqGHsislcqWGRbWAcqGGDbqxcqGHRaWkcqWGLbqzcqGGBbWwcqWGUbGBcqGGDbqxcqGGSaalaeaacqqGOaakcqWGUbGBcqGHLjYScqWGlbWscqGGPaqkaaGaeiilaWcaaa@5FBE@

where x˜
 MathType@MTEF@5@5@+=feaafiart1ev1aaatCvAUfKttLearuWrP9MDH5MBPbIqV92AaeXatLxBI9gBaebbnrfifHhDYfgasaacH8akY=wiFfYdH8Gipec8Eeeu0xXdbba9frFj0=OqFfea0dXdd9vqai=hGuQ8kuc9pgc9s8qqaq=dirpe0xb9q8qiLsFr0=vr0=vr0dc8meaabaqaciaacaGaaeqabaqabeGadaaakeaacuWG4baEgaacaaaa@2E34@[*n*] and *e*[*n*] represent the estimation of *x*[*n*] and the corresponding estimation error, respectively. Yeung et al. [31] have presented an application of the AR model to microarray gene expression time series analysis for gene regulation study. Comparing Equation (4) with Equation (5), it is obvious that Equation (5) is a special case of Equation (4).

For signal reconstruction in the shift-invariant space, we can characterize its energy density spectrum *S*_*xx*_(*ω*) according to Equation (4),. If *f*(*x*) ∈ *V *(*ϕ*), then the energy density spectrum is given by

Sxx(ω)=|∑kcke−i2πωkφ^(ω)|2
 MathType@MTEF@5@5@+=feaafiart1ev1aaatCvAUfKttLearuWrP9MDH5MBPbIqV92AaeXatLxBI9gBaebbnrfifHhDYfgasaacH8akY=wiFfYdH8Gipec8Eeeu0xXdbba9frFj0=OqFfea0dXdd9vqai=hGuQ8kuc9pgc9s8qqaq=dirpe0xb9q8qiLsFr0=vr0=vr0dc8meaabaqaciaacaGaaeqabaqabeGadaaakeaacqWGtbWudaWgaaWcbaGaemiEaGNaemiEaGhabeaakiabcIcaOGGaciab=L8a3jabcMcaPiabg2da9iabcYha8naawafabeWcbaGaem4AaSgabeqdbaGaeyyeIuoaaOGaem4yam2aaSbaaSqaaiabdUgaRbqabaGccqWGLbqzdaahaaWcbeqaaiabgkHiTiabdMgaPjabikdaYiab=b8aWjab=L8a3jabdUgaRbaakiqb=z8aMzaajaGaeiikaGIae8xYdCNaeiykaKIaeiiFaW3aaWbaaSqabeaacqaIYaGmaaaaaa@4F07@

where *ω *is the frequency in Hz. In order to avoid introducing spurious periodic components caused by a basis function *ϕ *with infinite support, for example, the sinc function, we only consider basis functions with compact support.

### PSD estimation algorithm

In [26], Grochenig and Schwab introduce the family of B-spline functions as the basis function with compact support. Assume that supp *ϕ *⊂[-Ω, Ω] and *f*(*x*) ∈ *V *(*ϕ*) is defined on a finite interval [*A*_1_, *A*_2_], then *f *can be determined completely by the coefficients {*c*_*k*_} for *k *∈ (*A*_1 _- Ω +1, *A*_2 _+ Ω -1) ∩ Z with

f(x)=∑k=A1−Ω+1A2+Ω−1ckφ(x−k)
 MathType@MTEF@5@5@+=feaafiart1ev1aaatCvAUfKttLearuWrP9MDH5MBPbIqV92AaeXatLxBI9gBaebbnrfifHhDYfgasaacH8akY=wiFfYdH8Gipec8Eeeu0xXdbba9frFj0=OqFfea0dXdd9vqai=hGuQ8kuc9pgc9s8qqaq=dirpe0xb9q8qiLsFr0=vr0=vr0dc8meaabaqaciaacaGaaeqabaqabeGadaaakeaacqWGMbGzcqGGOaakcqWG4baEcqGGPaqkcqGH9aqpdaGfWbqabSqaaiabdUgaRjabg2da9iabdgeabnaaBaaameaacqaIXaqmaeqaaSGaeyOeI0IaeuyQdCLaey4kaSIaeGymaedabaGaemyqae0aaSbaaWqaaiabikdaYaqabaWccqGHRaWkcqqHPoWvcqGHsislcqaIXaqma0qaaiabggHiLdaakiabdogaJnaaBaaaleaacqWGRbWAaeqaaGGacOGae8NXdyMaeiikaGIaemiEaGNaeyOeI0Iaem4AaSMaeiykaKcaaa@4E08@

In terms of the definition of the power spectrum density (PSD), we can obtain the following estimation function according to Equation (7)

Pxx(ω)=1A2−A1|∑k=A1−Ω+1A2+Ω−1cke−i2πωkφ^(ω)|2
 MathType@MTEF@5@5@+=feaafiart1ev1aaatCvAUfKttLearuWrP9MDH5MBPbIqV92AaeXatLxBI9gBaebbnrfifHhDYfgasaacH8akY=wiFfYdH8Gipec8Eeeu0xXdbba9frFj0=OqFfea0dXdd9vqai=hGuQ8kuc9pgc9s8qqaq=dirpe0xb9q8qiLsFr0=vr0=vr0dc8meaabaqaciaacaGaaeqabaqabeGadaaakeaacqWGqbaudaWgaaWcbaGaemiEaGNaemiEaGhabeaakiabcIcaOGGaciab=L8a3jabcMcaPiabg2da9maalaaabaGaeGymaedabaGaemyqae0aaSbaaSqaaiabikdaYaqabaGccqGHsislcqWGbbqqdaWgaaWcbaGaeGymaedabeaaaaGcdaabdaqaamaawahabeWcbaGaem4AaSMaeyypa0Jaemyqae0aaSbaaWqaaiabigdaXaqabaWccqGHsislcqqHPoWvcqGHRaWkcqaIXaqmaeaacqWGbbqqdaWgaaadbaGaeGOmaidabeaaliabgUcaRiabfM6axjabgkHiTiabigdaXaqdbaGaeyyeIuoaaOGaem4yam2aaSbaaSqaaiabdUgaRbqabaGccqWGLbqzdaahaaWcbeqaaiabgkHiTiabdMgaPjabikdaYiab=b8aWjab=L8a3jabdUgaRbaakiqb=z8aMzaajaGaeiikaGIae8xYdCNaeiykaKcacaGLhWUaayjcSdWaaWbaaSqabeaacqaIYaGmaaaaaa@639B@

Coefficients {*c*_*i*_} can be calculated according to following steps using least squares method [26]:

(1) Given sampling points *x*_1_,...,*x*_*J *_∈ [*A*_1_, *A*_2_] and corresponding discrete function values *y *= (*y*_1_,...,*y*_*J*_). Assume that *J *≥ *J*_min _= *A*_2 _- *A*_1 _+ 2Ω - 1 and that the truncated matrix *T *defined below is invertible. Compute matrix: *U *= (*U*_*jk*_), *T *= *T*_*kl*_), where

Ujk=φ(xj−k),Tkl=∑j=1Jφ(xj−k)¯φ(xj−l),
 MathType@MTEF@5@5@+=feaafiart1ev1aaatCvAUfKttLearuWrP9MDH5MBPbIqV92AaeXatLxBI9gBaebbnrfifHhDYfgasaacH8akY=wiFfYdH8Gipec8Eeeu0xXdbba9frFj0=OqFfea0dXdd9vqai=hGuQ8kuc9pgc9s8qqaq=dirpe0xb9q8qiLsFr0=vr0=vr0dc8meaabaqaciaacaGaaeqabaqabeGadaaakeaacqWGvbqvdaWgaaWcbaGaemOAaOMaem4AaSgabeaakiabg2da9GGaciab=z8aMjabcIcaOiabdIha4naaBaaaleaacqWGQbGAaeqaaOGaeyOeI0Iaem4AaSMaeiykaKIaeiilaWIaemivaq1aaSbaaSqaaiabdUgaRjabdYgaSbqabaGccqGH9aqpdaWfWaqaaiabggHiLdWcbaGaemOAaOMaeyypa0JaeGymaedabaGaemOsaOeaaOWaa0aaaeaacqWFgpGzcqGGOaakcqWG4baEdaWgaaWcbaGaemOAaOgabeaakiabgkHiTiabdUgaRjabcMcaPaaacqWFgpGzcqGGOaakcqWG4baEdaWgaaWcbaGaemOAaOgabeaakiabgkHiTiabdYgaSjabcMcaPiabcYcaSaaa@5993@

*j *= 1,...,*J*, *k*, *l *= *A*_1 _- Ω + 1,..., *A*_2 _+ Ω - 1.

(2) Compute *c *= *T*^-1^*b *according to *b *= U¯
 MathType@MTEF@5@5@+=feaafiart1ev1aaatCvAUfKttLearuWrP9MDH5MBPbIqV92AaeXatLxBI9gBaebbnrfifHhDYfgasaacH8akY=wiFfYdH8Gipec8Eeeu0xXdbba9frFj0=OqFfea0dXdd9vqai=hGuQ8kuc9pgc9s8qqaq=dirpe0xb9q8qiLsFr0=vr0=vr0dc8meaabaqaciaacaGaaeqabaqabeGadaaakeaadaqdaaqaaiabdwfavbaaaaa@2DF0@*y*, where U¯
 MathType@MTEF@5@5@+=feaafiart1ev1aaatCvAUfKttLearuWrP9MDH5MBPbIqV92AaeXatLxBI9gBaebbnrfifHhDYfgasaacH8akY=wiFfYdH8Gipec8Eeeu0xXdbba9frFj0=OqFfea0dXdd9vqai=hGuQ8kuc9pgc9s8qqaq=dirpe0xb9q8qiLsFr0=vr0=vr0dc8meaabaqaciaacaGaaeqabaqabeGadaaakeaadaqdaaqaaiabdwfavbaaaaa@2DF0@ denotes the complex conjugate transpose of *U *and *T*^-1 ^is the inverse of *T*.

**Remarks**:

(1) The construction of matrix *T *has the advantage that *T *is a positive operator on ℓ^2^(*Z*).

(2) In the case of B-spline, matrix *T *is invertible under the condition that

sup⁡j(xj+1−xj)≤(A2−A1)A2−A1+2Ω−1<1
 MathType@MTEF@5@5@+=feaafiart1ev1aaatCvAUfKttLearuWrP9MDH5MBPbIqV92AaeXatLxBI9gBaebbnrfifHhDYfgasaacH8akY=wiFfYdH8Gipec8Eeeu0xXdbba9frFj0=OqFfea0dXdd9vqai=hGuQ8kuc9pgc9s8qqaq=dirpe0xb9q8qiLsFr0=vr0=vr0dc8meaabaqaciaacaGaaeqabaqabeGadaaakeaacyGGZbWCcqGG1bqDcqGGWbaCdaWgaaWcbaGaemOAaOgabeaakiabcIcaOiabdIha4naaBaaaleaacqWGQbGAcqGHRaWkcqaIXaqmaeqaaOGaeyOeI0IaemiEaG3aaSbaaSqaaiabdQgaQbqabaGccqGGPaqkcqGHKjYOdaWcaaqaaiabcIcaOiabdgeabnaaBaaaleaacqaIYaGmaeqaaOGaeyOeI0Iaemyqae0aaSbaaSqaaiabigdaXaqabaGccqGGPaqkaeaacqWGbbqqdaWgaaWcbaGaeGOmaidabeaakiabgkHiTiabdgeabnaaBaaaleaacqaIXaqmaeqaaOGaey4kaSIaeGOmaiJaeuyQdCLaeyOeI0IaeGymaedaaiabgYda8iabigdaXaaa@525E@

That is, the Schoenberg-Whitney Theorem implies that *T *is invertible [32].

(3) Since the numerical solution of (*c*_*k*_) can be sensitive to particular sets of inter-sample spacing, we can approximate the inverse of the ill-conditioned *T *by its pseudo-inverse using singular value decomposition (SVD).

The B-spline of order *N *can be defined as the convolutions of (*N*+1) B-splines of order 0, i.e. φ=χ[−12,12]∗⋯∗χ[−12,12]︷N+1
 MathType@MTEF@5@5@+=feaafiart1ev1aaatCvAUfKttLearuWrP9MDH5MBPbIqV92AaeXatLxBI9gBaebbnrfifHhDYfgasaacH8akY=wiFfYdH8Gipec8Eeeu0xXdbba9frFj0=OqFfea0dXdd9vqai=hGuQ8kuc9pgc9s8qqaq=dirpe0xb9q8qiLsFr0=vr0=vr0dc8meaabaqaciaacaGaaeqabaqabeGadaaakeaaiiGacqWFgpGzcqGH9aqpdaagbaqaaiab=D8aJnaaBaaaleaacqGGBbWwcqGHsisldaWcaaqaaiabigdaXaqaaiabikdaYaaacqGGSaaldaWcaaqaaiabigdaXaqaaiabikdaYaaacqGGDbqxaeqaaOGaey4fIOIaeS47IWKaey4fIOIae83Xdm2aaSbaaSqaaiabcUfaBjabgkHiTmaalaaabaGaeGymaedabaGaeGOmaidaaiabcYcaSmaalaaabaGaeGymaedabaGaeGOmaidaaiabc2faDbqabaaabaGaemOta4Kaey4kaSIaeGymaedakiaawEJ=aaaa@4C52@. It is obvious that supp φ⊂[−N+12,N+12]
 MathType@MTEF@5@5@+=feaafiart1ev1aaatCvAUfKttLearuWrP9MDH5MBPbIqV92AaeXatLxBI9gBaebbnrfifHhDYfgasaacH8akY=wiFfYdH8Gipec8Eeeu0xXdbba9frFj0=OqFfea0dXdd9vqai=hGuQ8kuc9pgc9s8qqaq=dirpe0xb9q8qiLsFr0=vr0=vr0dc8meaabaqaciaacaGaaeqabaqabeGadaaakeaaiiGacqWFgpGzcqGHckcZcqGGBbWwcqGHsisldaWcbaWcbaGaemOta4Kaey4kaSIaeGymaedabaGaeGOmaidaaOGaeiilaWYaaSqaaSqaaiabd6eaojabgUcaRiabigdaXaqaaiabikdaYaaakiabc2faDbaa@3CD3@ for a B-spline of order *N *and its Fourier transform can be easily computed as follows:

φ^(ω)=[sin⁡(πω)πω]N+1
 MathType@MTEF@5@5@+=feaafiart1ev1aaatCvAUfKttLearuWrP9MDH5MBPbIqV92AaeXatLxBI9gBaebbnrfifHhDYfgasaacH8akY=wiFfYdH8Gipec8Eeeu0xXdbba9frFj0=OqFfea0dXdd9vqai=hGuQ8kuc9pgc9s8qqaq=dirpe0xb9q8qiLsFr0=vr0=vr0dc8meaabaqaciaacaGaaeqabaqabeGadaaakeaaiiGacuWFgpGzgaqcaiabcIcaOiab=L8a3jabcMcaPiabg2da9maadmaabaWaaSaaaeaacyGGZbWCcqGGPbqAcqGGUbGBdaqadaqaaiab=b8aWjab=L8a3bGaayjkaiaawMcaaaqaaiab=b8aWjab=L8a3baaaiaawUfacaGLDbaadaahaaWcbeqaaiabd6eaojabgUcaRiabigdaXaaaaaa@44D9@

Substituting it to (8), we obtain an explicit formulation of the PSD estimation as follows:

Pxx(ω)=1A2−A1|∑k=A1−Ω+1A2+Ω−1cke−i2πωk[sin⁡(πω)πω]N+1|2
 MathType@MTEF@5@5@+=feaafiart1ev1aaatCvAUfKttLearuWrP9MDH5MBPbIqV92AaeXatLxBI9gBaebbnrfifHhDYfgasaacH8akY=wiFfYdH8Gipec8Eeeu0xXdbba9frFj0=OqFfea0dXdd9vqai=hGuQ8kuc9pgc9s8qqaq=dirpe0xb9q8qiLsFr0=vr0=vr0dc8meaabaqaciaacaGaaeqabaqabeGadaaakeaacqWGqbaudaWgaaWcbaGaemiEaGNaemiEaGhabeaakiabcIcaOGGaciab=L8a3jabcMcaPiabg2da9maalaaabaGaeGymaedabaGaemyqae0aaSbaaSqaaiabikdaYaqabaGccqGHsislcqWGbbqqdaWgaaWcbaGaeGymaedabeaaaaGcdaabdaqaamaawahabeWcbaGaem4AaSMaeyypa0Jaemyqae0aaSbaaWqaaiabigdaXaqabaWccqGHsislcqqHPoWvcqGHRaWkcqaIXaqmaeaacqWGbbqqdaWgaaadbaGaeGOmaidabeaaliabgUcaRiabfM6axjabgkHiTiabigdaXaqdbaGaeyyeIuoaaOGaem4yam2aaSbaaSqaaiabdUgaRbqabaGccqWGLbqzdaahaaWcbeqaaiabgkHiTiabdMgaPjabikdaYiab=b8aWjab=L8a3jabdUgaRbaakmaadmaabaWaaSaaaeaacyGGZbWCcqGGPbqAcqGGUbGBdaqadaqaaiab=b8aWjab=L8a3bGaayjkaiaawMcaaaqaaiab=b8aWjab=L8a3baaaiaawUfacaGLDbaadaahaaWcbeqaaiabd6eaojabgUcaRiabigdaXaaaaOGaay5bSlaawIa7amaaCaaaleqabaGaeGOmaidaaaaa@7044@

We have the following observations on our PSD estimation method:

• The proposed method does not make any assumption about the number of frequency components in the data, whereas the Lomb-Scargle method assumes that there is only a single stationary sinusoid wave with infinite support. In practice, noise in the data can contribute to many frequency components. Thus, our model is more appropriate for real-world noisy data.

• Compared with the traditional PSD estimation algorithms, our method can directly compute the PSD for an unevenly sampled signal from Equation (11). The classical Fourier periodogram based method only can deal with evenly sampled data.

• The order *N *of the B-spline basis function *ϕ *can be chosen adaptively according to Equation (9). From Equation (9), we know that the relationship between the B-spline order *N *and the minimum number of sampling points *J*_min _is given by *N *≈ *J*_min _- (*A*_2 _- *A*_1_) such that the matrix T is invertible. If we hold the number of sampling point *J *and the signal support [*A*_1_, *A*_2_] fixed, then increasing *N *(which would result in a smoother B-spline with larger support) would ensures a smoother reconstruction. In practice, some over-sampling is done for stability of reconstruction, and we increase the over-sampling rate (i.e., number of sample time points per time interval) by time-scaling the original signal support appropriately to a smaller interval.

• From Equation (11), we see that the periodogram decays more rapidly with increasing *N*, meaning that the reconstructed signal will be smoother with increasing *N*. This fact can be used for signal denoising.

### Detecting periodically expressed genes

The periodogram of the microarray time series from a periodically expressed gene must contain a peak corresponding to its dominant frequency. In [27], Bozdech et al. use the power ratio test to detect the periodic genes of Plasmodium falciparum. Assuming that the frequency value of the main peak in the periodogram is *f*_*m *_and consider the frequency band [*f*_*m*-1_, *f*_*m*+1_] as *f*_*m*_'s region of influence (ROI). The power ratio *S *in [27] is defined to be the ratio of the power in *f*_*m*_'s ROI, *power*_*m*_, to the total power of the signal, *power*_*total*_, i.e.,:

S=powermpowertotal
 MathType@MTEF@5@5@+=feaafiart1ev1aaatCvAUfKttLearuWrP9MDH5MBPbIqV92AaeXatLxBI9gBaebbnrfifHhDYfgasaacH8akY=wiFfYdH8Gipec8Eeeu0xXdbba9frFj0=OqFfea0dXdd9vqai=hGuQ8kuc9pgc9s8qqaq=dirpe0xb9q8qiLsFr0=vr0=vr0dc8meaabaqaciaacaGaaeqabaqabeGadaaakeaacqWGtbWucqGH9aqpdaWcaaqaaiabdchaWjabd+gaVjabdEha3jabdwgaLjabdkhaYnaaBaaaleaacqWGTbqBaeqaaaGcbaGaemiCaaNaem4Ba8Maem4DaCNaemyzauMaemOCai3aaSbaaSqaaiabdsha0jabd+gaVjabdsha0jabdggaHjabdYgaSbqabaaaaaaa@45B9@

If the power ratio calculated is larger than a threshold, the corresponding profile is considered to be periodic. In [27], the threshold for *S *is heuristically chosen to be 0.7.

In order to be rigorous statistically, Chen [10] and Wichert et al. [11] use the Fisher G-statistic test to determine whether a peak in the periodogram is significant or not. They corrected for the multiple testing case using the method of False Discovery Rate (FDR) [33], which controls the expected proportion of false positives in the result. The Fisher G-statistic test [34] can be used to test for the presence of periodic component in random white noise for finite sample signal. The *p*-value for the hypothesis testing for periodicity of a signal g of length *N*_0_, using G-statistic as the test statistic, is given by

P(g>x)=∑j=1p(−1)j−1(nj)(1−jx)n−1
 MathType@MTEF@5@5@+=feaafiart1ev1aaatCvAUfKttLearuWrP9MDH5MBPbIqV92AaeXatLxBI9gBaebbnrfifHhDYfgasaacH8akY=wiFfYdH8Gipec8Eeeu0xXdbba9frFj0=OqFfea0dXdd9vqai=hGuQ8kuc9pgc9s8qqaq=dirpe0xb9q8qiLsFr0=vr0=vr0dc8meaabaqaciaacaGaaeqabaqabeGadaaakeaacqWGqbaucqGGOaakcqWGNbWzcqGH+aGpcqWG4baEcqGGPaqkcqGH9aqpdaaeWbqaaiabcIcaOiabgkHiTiabigdaXiabcMcaPmaaCaaaleqabaGaemOAaOMaeyOeI0IaeGymaedaaOWaaeWaaeaafaqabeGabaaabaGaemOBa4gabaGaemOAaOgaaaGaayjkaiaawMcaaiabcIcaOiabigdaXiabgkHiTiabdQgaQjabdIha4jabcMcaPmaaCaaaleqabaGaemOBa4MaeyOeI0IaeGymaedaaaqaaiabdQgaQjabg2da9iabigdaXaqaaiabdchaWbqdcqGHris5aaaa@5084@

where *g *is the sample realization of the G-statistic value calculated from the Fisher's G-statistic given by

g=max⁡kPxx(ωk)∑k=1nPxx(ωk)
 MathType@MTEF@5@5@+=feaafiart1ev1aaatCvAUfKttLearuWrP9MDH5MBPbIqV92AaeXatLxBI9gBaebbnrfifHhDYfgasaacH8akY=wiFfYdH8Gipec8Eeeu0xXdbba9frFj0=OqFfea0dXdd9vqai=hGuQ8kuc9pgc9s8qqaq=dirpe0xb9q8qiLsFr0=vr0=vr0dc8meaabaqaciaacaGaaeqabaqabeGadaaakeaacqWGNbWzcqGH9aqpdaWcaaqaaiGbc2gaTjabcggaHjabcIha4naaBaaaleaacqWGRbWAaeqaaOGaemiuaa1aaSbaaSqaaiabdIha4jabdIha4bqabaGccqGGOaakiiGacqWFjpWDdaWgaaWcbaGaem4AaSgabeaakiabcMcaPaqaamaaqadabaGaemiuaa1aaSbaaSqaaiabdIha4jabdIha4bqabaGccqGGOaakcqWFjpWDdaWgaaWcbaGaem4AaSgabeaakiabcMcaPaWcbaGaem4AaSMaeyypa0JaeGymaedabaGaemOBa4ganiabggHiLdaaaaaa@4E5C@

*n *= [*N*_0_/2], and *p *is the largest integer less than 1/*x*. Equation (14) is computed over the set of normalized Fourier frequencies *ω *= *k*/*N*_0_, where *k *= 0,1,...,*N*_0_/2. Equation (13) gives an exact *p*-value that allows one to test whether a gene expression profile behaves like a purely random white noise process or whether the maximum peak in the periodogram is significant. However, for gene expression profiles of short length, i.e., < 40 time points, the p-value given by Equation (13) has weak statistical power to determine the number of periodic genes [9,11]. In view of this, we follow [28] and provide instead a ranking of the expression profiles based on the G-statistic of Equation (14).

### Lomb-Scargle method

Here, we briefly review the Lomb-Scargle periodogram method. For a time series *X*(*t*_*j*_), where *i *= 1,2,...,*N*_0 _and zero mean, the normalized periodogram as a function of the angular frequency *ω *is defined as [16]

Pxx(ω)=12σ2{[∑j=1N0X(tj)cos⁡ω(tj−τ)]2∑j=1N0cos⁡2ω(tj−τ)+[∑j=1N0X(tj)sin⁡ω(tj−τ)]2∑j=1N0sin⁡2ω(tj−τ)}
 MathType@MTEF@5@5@+=feaafiart1ev1aaatCvAUfKttLearuWrP9MDH5MBPbIqV92AaeXatLxBI9gBaebbnrfifHhDYfgasaacH8akY=wiFfYdH8Gipec8Eeeu0xXdbba9frFj0=OqFfea0dXdd9vqai=hGuQ8kuc9pgc9s8qqaq=dirpe0xb9q8qiLsFr0=vr0=vr0dc8meaabaqaciaacaGaaeqabaqabeGadaaakeaacqWGqbaudaWgaaWcbaGaemiEaGNaemiEaGhabeaakiabcIcaOGGaciab=L8a3jabcMcaPiabg2da9maalaaabaGaeGymaedabaGaeGOmaiJae83Wdm3aaWbaaSqabeaacqaIYaGmaaaaaOWaaiWaaeaadaWcaaqaamaadmaabaWaaabmaeaacqWGybawcqGGOaakcqWG0baDdaWgaaWcbaGaemOAaOgabeaakiabcMcaPiGbcogaJjabc+gaVjabcohaZjab=L8a3jabcIcaOiabdsha0naaBaaaleaacqWGQbGAaeqaaOGaeyOeI0Iae8hXdqNaeiykaKcaleaacqWGQbGAcqGH9aqpcqaIXaqmaeaacqWGobGtdaWgaaadbaGaeGimaadabeaaa0GaeyyeIuoaaOGaay5waiaaw2faamaaCaaaleqabaGaeGOmaidaaaGcbaWaaabmaeaacyGGJbWycqGGVbWBcqGGZbWCdaahaaWcbeqaaiabikdaYaaakiab=L8a3jabcIcaOiabdsha0naaBaaaleaacqWGQbGAaeqaaOGaeyOeI0Iae8hXdqNaeiykaKcaleaacqWGQbGAcqGH9aqpcqaIXaqmaeaacqWGobGtdaWgaaadbaGaeGimaadabeaaa0GaeyyeIuoaaaGccqGHRaWkdaWcaaqaamaadmaabaWaaabmaeaacqWGybawcqGGOaakcqWG0baDdaWgaaWcbaGaemOAaOgabeaakiabcMcaPiGbcohaZjabcMgaPjabc6gaUjab=L8a3jabcIcaOiabdsha0naaBaaaleaacqWGQbGAaeqaaOGaeyOeI0Iae8hXdqNaeiykaKcaleaacqWGQbGAcqGH9aqpcqaIXaqmaeaacqWGobGtdaWgaaadbaGaeGimaadabeaaa0GaeyyeIuoaaOGaay5waiaaw2faamaaCaaaleqabaGaeGOmaidaaaGcbaWaaabmaeaacyGGZbWCcqGGPbqAcqGGUbGBdaahaaWcbeqaaiabikdaYaaakiab=L8a3jabcIcaOiabdsha0naaBaaaleaacqWGQbGAaeqaaOGaeyOeI0Iae8hXdqNaeiykaKcaleaacqWGQbGAcqGH9aqpcqaIXaqmaeaacqWGobGtdaWgaaadbaGaeGimaadabeaaa0GaeyyeIuoaaaaakiaawUhacaGL9baaaaa@A5C7@

where *τ *is defined by the equation

tan⁡(2ωτ)=(∑j=1N0sin⁡2ωtj)/(∑j=1N0cos⁡2ωtj)
 MathType@MTEF@5@5@+=feaafiart1ev1aaatCvAUfKttLearuWrP9MDH5MBPbIqV92AaeXatLxBI9gBaebbnrfifHhDYfgasaacH8akY=wiFfYdH8Gipec8Eeeu0xXdbba9frFj0=OqFfea0dXdd9vqai=hGuQ8kuc9pgc9s8qqaq=dirpe0xb9q8qiLsFr0=vr0=vr0dc8meaabaqaciaacaGaaeqabaqabeGadaaakeaacyGG0baDcqGGHbqycqGGUbGBcqGGOaakcqaIYaGmiiGacqWFjpWDcqWFepaDcqGGPaqkcqGH9aqpcqGGOaakdaaeWbqaaiGbcohaZjabcMgaPjabc6gaUjabikdaYiab=L8a3jabdsha0naaBaaaleaacqWGQbGAaeqaaaqaaiabdQgaQjabg2da9iabigdaXaqaaiabd6eaonaaBaaameaacqaIWaamaeqaaaqdcqGHris5aOGaeiykaKIaei4la8IaeiikaGYaaabCaeaacyGGJbWycqGGVbWBcqGGZbWCcqaIYaGmcqWFjpWDcqWG0baDdaWgaaWcbaGaemOAaOgabeaaaeaacqWGQbGAcqGH9aqpcqaIXaqmaeaacqWGobGtdaWgaaadbaGaeGimaadabeaaa0GaeyyeIuoakiabcMcaPaaa@5FB6@

The statistical significance of the periodic components detected in the periodogram can be evaluated using an exponential probability distribution test [16] which we denoted in this work as the Lomb-Scargle test.

Authors' contributionsAWCL worked on mathematical modelling, implementation, and microarray datasets analysis. JX worked on signal reconstruction theories and is responsible for the simulated signal experiments. SHW worked on signal reconstruction theories. DS provided biological insight to this project. HY initiated the project and worked on spectral estimation. All authors read and approved the final manuscript.

## Supplementary Material

Additional File 1 **Ranked list of 6875 profiles of the Plasmodium falciparum dataset based on the G-statistic**. A ranked list of the 6875 profiles from the Plasmodium falciparum dataset [27] based on their G-statistics. The higher the rank, the more periodic is the profile. Click here for file

Additional File 2 **Ranked lists of 6178 profiles of the Yeast dataset based on the G-statistic in the alpha factor, cdc15, cdc28, and elutriation experiments**. A ranked list of the 6178 yeast expression profiles in each of the four experiments (alpha factor, cdc15, cdc28, and elutriation) based on their G-statistics. The higher the rank, the more periodic is the profile. Click here for file
